# Potential Target Antigens for a Universal Vaccine in Epithelial Ovarian Cancer

**DOI:** 10.1155/2010/891505

**Published:** 2010-09-15

**Authors:** Renee Vermeij, Toos Daemen, Geertruida H. de Bock, Pauline de Graeff, Ninke Leffers, Annechien Lambeck, Klaske A. ten Hoor, Harry Hollema, Ate G. J. van der Zee, Hans W. Nijman

**Affiliations:** ^1^Department of Gynecological Oncology, University Medical Center Groningen, University of Groningen, P.O. Box 30.001, 9700 RB Groningen, The Netherlands; ^2^Department of Medical Microbiology, Molecular Virology Section, University Medical Center Groningen, University of Groningen, P.O. Box 30.001, 9700 RB Groningen, The Netherlands; ^3^Department of Epidemiology, University Medical Center Groningen, University of Groningen, P.O. Box 30.001, 9700 RB Groningen, The Netherlands; ^4^Department of Pathology and Medical Biology, University Medical Center Groningen, University of Groningen, P.O. Box 30.001, 9700 RB Groningen, The Netherlands

## Abstract

The prognosis of epithelial ovarian cancer (EOC), the primary cause of death from gynaecological malignancies, has only modestly improved over the last decades. Immunotherapeutic treatment using a cocktail of antigens has been proposed as a “universal” vaccine strategy. We determined the expression of tumor antigens in the context of MHC class I expression in 270 primary tumor samples using tissue microarray. Expression of tumor antigens p53, SP17, survivin, WT1, and NY-ESO-1 was observed in 120 (48.0%), 173 (68.9%), 208 (90.0%), 129 (56.3%), and 27 (11.0%) of 270 tumor specimens, respectively. In 93.2% of EOC, at least one of the investigated tumor antigens was (over)expressed. Expression of MHC class I was observed in 78.1% of EOC. In 3 out 4 primary tumors, (over)expression of a tumor antigen combined with MHC class I was observed. These results indicate that a multiepitope vaccine, comprising these antigens, could serve as a universal therapeutic vaccine for the vast majority of ovarian cancer patients.

## 1. Introduction

Epithelial ovarian cancer (EOC) is the most common cause of death in gynaecologic malignancies [1]. Most ovarian cancer patients are asymptomatic until disease has metastasized and therefore two-thirds of all patients are diagnosed with advanced stage disease [1, 2]. Although the majority of patients with advanced disease achieve complete clinical response rates due to the current therapy of aggressive cytoreductive surgery and platinum-taxane-based chemotherapy, more than 90% develop tumor recurrence, resulting in five-year survival rates of only 30% [3]. 

These records express the need for a new and improved therapy for EOC. The significance of the immune response for the clinical course of EOC has led to attempts to modulate it artificially with (antigen-specific) immunotherapeutic strategies [4]. Presentation of tumor antigens in the context of MHC molecules on tumor cells is critical for the efficacy of targeted immunotherapy [5]. Thus far, approaches at therapeutic vaccination in cancer patients including administration of peptide pulsed dendritic cells, recombinant viral vectors encoding tumor antigen, DNA-fusion vaccine and single peptide vaccine have not shown consistent, and high percentages of clinical successes [6–12]. Most clinical studies on immunotherapy targeted one antigen, limiting the use of such vaccines to those patients with (over)expression of that specific tumor antigen. Immunization using a cocktail of antigens has been proposed as a “universal” vaccine strategy. Whereas solid tumors often show heterogeneous protein expression, multiantigen vaccines may have greater therapeutic potential and compensate for tumor antigen-loss variants [13, 14]. The ability to target multiple antigens, may also improve the immunogenicity of therapeutic vaccines [13, 15]. Therefore, discovery of multiple tumor antigens in EOC may provide opportunities for multiantigen immunotherapeutic strategies that can induce sufficient clinical responses. Tumor antigens that are inherently immunogenic and oncogenic in ovarian cancer are p53 [16–18], Sperm Protein 17 (SP17) [14, 19, 20], Wilms' tumor gene 1 (WT1) [21–23], survivin [24–26], and NY-ESO-1 [12, 27, 28]. 

The presence of an *α* (heavy) chain and *β*_2_-microglobulin is a prerequisite for the formation of a stable MHC class I complex [29]. Such stable MHC class I complexes are required for presentation of the tumor antigenic peptides [30]. 

No reports have been published describing tissue microarray staining of p53, SP17, survivin, WT1, and NY-ESO-1 with MHC class I expression in EOC. Further knowledge of the expression of multiple tumor antigens in the context of MHC class I expression is necessary to develop strategies to increase clinical efficacy of multiantigen immunotherapy in EOC. 

The aim of the present study was to investigate the expression of SP17 and NY-ESO-1 and overexpression of p53, WT1, and survivin together with *β*_2_-microglobulin and the *α*-chains, HLA-A and HLA-B/C, in tumor samples obtained from a large well-documented cohort of primary EOC patients using tissue microarray.

## 2. Materials and Methods

### 2.1. Patients

Since 1985, the Department of Gynecological Oncology of the University Medical Centre Groningen (UMCG) prospectively stores all clinicopathologic and followup data of epithelial ovarian cancer patients in a computerized database. Tumor samples from 361 patients were collected on a tissue microarray. This tissue microarray contains primary ovarian tumor tissue obtained before chemotherapy of 270 patients. Patients with borderline or nonepithelial tumors were excluded. Primary treatment for all patients consisted of surgery and adjuvant chemotherapeutic treatment consistent of platinum-based regimens and others. Since 1995, platinum-based chemotherapy was supplemented by taxanes. 

In the current study, the 270 EOC patients were selected for tumor antigen analysis who underwent primary surgery between 1985 and 2006 and of whom sufficient paraffin-embedded ovarian tumor tissue and complete followup data were available. In a nonselected subgroup of 183 primary EOC patients, MHC class I expression was analyzed. These data are partly previously published by our group [29].

Patients were surgically staged according to FIGO (International Federation of Gynecology and Obstetrics) classification [31]. Optimal and suboptimal debulking was defined as the largest residual tumor lesions having a diameter of, respectively, <2 cm or ≥2 cm. Histology of all tumors was determined according to World Health Organization criteria [32].

All relevant data were filed in a separate anonymous database in which patients were given unique codes to protect patient identity. Database management was restricted to two people with access to the larger database containing all patients' characteristics. Due to these procedures, no additional patient or institutional review board approval was required according to Dutch Law.

### 2.2. Tissue Microarrays

Tissue microarrays were constructed as described previously [17]. Four cores of 0.6 mm² were taken by biopsy and placed by a tissue microarrayer (Beecher Instruments, Silver Spring, MD, USA) on a recipient paraffin block. Using a microtome, 4 *μ*m sections were cut from each tissue microarray block and applied to aminopropyltriethoxysilane-coated slides. All arrayed samples were H&E-stained to confirm the presence of tumor tissue.

### 2.3. Immunohistochemical Staining of Tissue Microarrays

Tissue microarray sections were deparaffinized in xylene and rehydrated through graded concentrations of ethanol to distilled water. The sections were boiled for 15 minutes in a microwave to accomplish antigen retrieval. Endogenous peroxidase was blocked by incubation of sections for 30 minutes in 0.3% hydrogen peroxide. Primary antibodies, antigen retrieval buffers, and detection methods used are provided as supplementary data (Table 1). Sections were counterstained with hematoxylin. All control experiments gave satisfactory results.

### 2.4. Scoring

Evaluation of immunostaining was independently performed by two observers blinded to the clinical data. Agreement between the two observers was >90%. Contradictory outcomes were reviewed by a gynecological pathologist and were reassigned by approval of all parties. 

Immunostaining for p53, HLA-A, HLA-B/C, and *β*_2_-m was scored as described in previous studies [17, 29, 30]. The immunohistochemical reaction for SP17 [33], WT1 [34, 35], survivin [24, 36, 37], and NY-ESO-1 [38] was semiquantitatively graded into four classes based on the frequency of nuclear staining for SP17, WT1, and survivin, and cytoplasmatic staining in NY-ESO-1 in ovarian cancer cells: negative = no/very low frequency (<5%) immunopositive cells; + = low frequency (≤5–25%); ++ = moderate frequency (25%–50%); +++ = high frequency (50%–75%); ++++ = very high frequency (75–100%). The cutoff was `a priori` chosen for scoring; cases with low frequency or higher were considered positive for tumor antigen expression.

### 2.5. Statistical Analysis of Data

Statistical analysis was carried out using the SPSS 16.0 software package fow Windows (SPSS Inc., Chicago, USA). All cases with <2 evaluable cores were excluded from analysis. 

## 3. Results

### 3.1. Patients

Tumor samples from 270 consecutive primary ovarian cancer patients (median age 56.9 years, range 16–89) treated at the UMCG between 1985 and 2006 were available (Table 2). The majority of patients presented with serous histology, advanced stage, and/or high-grade disease. First-line chemotherapy regimens were platinum based in 90 (34.2%) patients and platinum and taxane based in 108 (41.1%) patients. Other regimens were given to 25 (9.5%) patients, while 40 (15.2%) patients did not receive chemotherapy because of early stage disease, comorbidity, or treatment refusal.

### 3.2. Tumor Antigen (Over)Expression in EOC

P53, SP17, survivin, WT1, and NY-ESO-1 (over)expression was observed in 48.0%, 68.9%, 90.0%, 56.3%, and 11.0% of tumors, respectively (Table 3). In 93.2% tumors, at least one of the investigated tumor antigens was (over)expressed (Table 4). Expression of only one tumor antigen was found in 40 (15.2%) tumors, 70 (26.6%) tumors expressed two antigens, 70 (26.6%) tumors expressed three antigens, 58 (22.1%) tumors expressed four antigens, and 7 (2.7%) tumors expressed all five investigated tumor antigens. Absence of expression of any antigen was seen in 18 (6.8%) patients. Nonevaluable primary tumors due to core loss during staining procedures or absence of tumor tissue ranged from 19 (7.4%) for SP17 staining to 41 (15.2%) for WT1 staining. Several specific combinations of tumor antigen expression cover high percentages of EOC patients, varying from 95.5% (214/224) combining two antigens to a maximum coverage of 98.2% (216/220) combining four antigens (Table 5).

### 3.3. Immunostaining MHC Class I

Coexpression of HLA-A and *β*_2_-m or HLA-B/C and *β*_2_-m was observed in 98 (53.6%) and 136 (74.7%) of the tumors, respectively (Table 3). Positive MHC class I expression, defined as HLA-A and *β*_2_-m and/or HLA-B/C and *β*_2_-m coexpression, was observed in 143 (78.1%) tumors.

### 3.4. Coexpression of Tumorantigens and MHC Class I in EOC

Of all EOC positive for p53, SP17, survivin, WT1, or NY-ESO-1, 82.5%, 82.8%, 77.0%, 80.9%, and 80.0% were also positive for MHC class I, respectively (Table 6). In 78.4% of tumors (over)expressing one or more tumor antigens, also expression of MHC class I was found. Furthermore, 74.3% of all tumors coexpressed MHC class I and at least one tumor antigen.

## 4. Discussion

In a large well-documented cohort of representative EOC patients, (over)expression of at least one of the tumor antigens p53, SP17, survivin, WT1, or NY-ESO-1 was observed in over 90% of the tumors. To our knowledge, this is the first study on the expression of multiple tumor antigens in a large cohort of EOC. Only a minority (6.8%) of the tumors did not express one of the selected tumor antigens. About 75% of the EOC tumors expressed both, one of the tumor antigens and MHC class I. This observation underlines the relevance of designing a multiepitope vaccine consisting of p53, SP17, NY-ESO-1, survivin, and WT1 for the immunotherapeutic treatment of ovarian cancer.

This inventory tissue microarray study enables us to analyze the expression of five well-known tumor antigens in EOC, in correlation to MHC class I expression. Tissue microarray is a practical and powerful tool for high-throughput analysis of tumor tissue identifying targets in human cancers [39]. P53, SP17, NY-ESO-1, survivin, and WT1 are immunogenic target antigens in EOC. Rates of observed (over)expression of p53, SP17, survivin, and WT1 in 48.0%, 68.9%, 90.0%, and 56.3% of EOC patients, respectively, are in agreement with previous studies [16, 24, 40, 41]. NY-ESO-1 expression was seen in 11.0% of tumors in our cohort which is in agreement with the results of others [42, 43]. However, Odunsi *et al*. observed NY-ESO-1 expression in 43% of EOC patients [38, 44]. This difference in expression might be explained by considerable methodological variability among the different studies. The type of study design, antibodies and assays used to study NY-ESO-1 expression, determination of cutoff points for aberrant NY-ESO-1 expression, and the definition of study end points vary greatly among different studies. Immunohistochemical analyses of tumors have shown heterogeneous NY-ESO-1 expression [45]. Since expression of NY-ESO-1 is mostly focal and nonuniform, tissue microarrays containing large numbers of tumor tissue are essential to determine NY-ESO-1 expression in EOC. Our sample size of 270 EOC patients might be more potent to distinguish between positive and negative NY-ESO-1 expression in EOC compared to 143 EOC patients analyzed by Odunsi *et al*.

We previously reported on the expression of tumor antigens EGFR and Her-2 in our large well-documented cohort of representative EOC, using tissue microarray [46]. EGFR and Her-2 overexpression was observed in 7.0% and 5.2% of EOC, respectively. The expression of EGFR and Her-2 has been extensively studied in ovarian cancer [47, 48]. Aberrant activity of these antigens is important in tumor growth and development [49, 50]. Therefore, EGFR and Her-2 were considered to be attractive targets for immunotherapeutic strategies in EOC. Because of the low expression levels in EOC, therapeutic potential of vaccines targeting EGFR and Her-2 is limited. As the existing repertoire of known antigens in EOC is relatively small, we performed our innovative study on five highly expressed tumor antigens which may provide opportunities for multiepitope immunotherapeutic strategies targeting the majority of EOC patients. 

We provide first evidence that several antigen combinations can be used in a multiepitope vaccine for EOC treatment, since different antigen combinations cover high percentages of EOC patients. Vaccines comprising a mixture of, for example, p53, SP17, and survivin or combining survivin, WT1, and NY-ESO-1 cover the vast majority of EOC patients. Maximum coverage of EOC patients can be obtained by a vaccine comprising four antigens p53, SP17, survivin, and WT1. 

Single antigen vaccines targeting p53 [51], SP17 [40], NY-ESO-1 [52], survivin [11], and WT1 [22] have been described to generate tumor antigen-specific cytotoxic T-cell lymphocytes (CTLs) able to lyse autologous tumor cells. One can envision that multiepitope vaccines may enhance immunogenicity, improving clinical efficacy of the immunotherapeutic vaccine.

Multiepitope vaccines should preferably contain multiple MHC class I-presented CTL epitopes derived from different target antigens together with a tumor-specific MHC class II-presented T-helper epitope. This will reduce the risk of immune-driven selection of antigen-loss variants of the tumor. Next, given the pivotal role of T-helper cells in promoting the primary and secondary CTL responses through the induction of DC maturation and the production of cytokines, the inclusion of T-helper epitopes in a multiepitope-based vaccine will have strong beneficial effects [6]. For example, p53-specific T-helper cells induced upon p53 specific immunization might fulfil this role [53]. 

Important advantages of well-defined multiepitope vaccines over nondefined vaccines, such as tumor lysate vaccines, are their defined nature [6, 54], lack of suppressive inducing antigens [55–57], simple way of manipulation to prevent dominance of one antigen over the others [6, 58], universal applicability [6, 59], easiness to make in a standardized procedure [59, 60], possibility to combine with other strategies [59], and limited autoimmune toxicity [55, 61]. 

Moreover, administration of a multiepitope vaccine as a single mixture offers advantages including: (1) injection of a limited volume, (2) lower number of skin sites with local toxicity due to injection site reactions, and (3) lower chance of error and contamination with the preparation of one versus multiple epitope preparations [13, 14].

In contrast, previous studies showed that administration of multiple epitopes at one injection site could lead to a more vigorous response to just one of the involved antigens [62, 63]. We reasoned that this disadvantageous result might be due to immunodominance of one antigen over the other. Preclinical studies might be helpful in designing the optimal combination of multiantigen vaccines, trying to predict and/or prevent immunodominance. In contrast, the synergy between antigens included in a multiepitope vaccine might induce immune responses with increased potency compared with the response induced by the same epitopes individually [6]. Separate injection sites for all of the involved antigens may result in a significant increase in the magnitude of the antigen-specific T-cell response. It still holds true that several multiantigen combinations cover high percentages of tumors. The most favourable vaccine, based on (pre)clinical studies concerning immunodominance, can be used for treatment of EOC patients. 

MHC class I downregulation was observed in 21.9% of tumors. Loss of MHC class I molecules on tumor cells, which may lead to immune escape, is often restricted to one or a few alleles. Targeting multiple epitopes restricted by different class I molecules of the patient will likely circumvent such an escape mechanism [6]. The tumor-associated antigens p53, NY-ESO-1, and WT1 epitopes are presented both by MHC class I and II (according to listing at http://www.cancerimmunity.org/, update September 2008). As a result, p53, WT1, and NY-ESO-1 [64] can function as both CTL and T-helper cell targets. 

Considering the importance of the expression of MHC class I by tumor cells for immune recognition by T cells, several regimens could be added in the multiepitope vaccine to enhance MHC class I expression. Treatment with IFN-gamma is known to upregulate MHC class I [10, 65, 66]. Another possibility would be the addition of demethylating agents to the multiepitope vaccine, since DNA hypermethylation, common in human tumors, may result in the loss of MHC class I expression [67, 68].

The most promising finding that emerges from this study is that the vast majority of EOC patients present one or more tumor antigens. Furthermore, if tumor cells present one of our investigated tumor antigens, it is likely to express MHC class I as well. Therefore, a vaccine comprising the investigated tumor antigens is capable of targeting tumor cells of the vast majority of EOC patients. Since several combinations of tumor antigens cover the majority of EOC patients, different institutes can attribute personally preferred antigens to their multiepitope vaccine.

In summary, we are first to show that multiepitope immunotherapy combining tumor antigens p53, SP17, survivin, WT1, and/or NY-ESO-1 might be a promising new therapeutic vaccination strategy in ovarian cancer.

## Figures and Tables

**Table 1:** Antibodies used for immunohistochemical staining.

Antigen	Antigen retrieval	Clone	Dilution	Company
p53	Tris/EDTA (pH8)	DO-7^1^	1 : 2000	DAKO^2^
SP17	Citrate (pH 6)	Sp17MF1	1 : 100	^3^
survivin	Citrate (pH 6)	71G4B7E	1 : 100	Cell signaling^4^
WT1	Tris/HCL (pH 9)	6F-H2	1 : 25	DAKO^2^
NY-ESO-1	EDTA (pH 8)	E978	1 : 50	Zymed^5^
HLA-A	Citrate (pH 6)	HCA2	1 : 500	^6^
HLA-B/C	Citrate (pH 6)	HC-10	1 : 100	^6^
*β*_2_-m	Citrate (pH 6)	Polyclonal	1 : 400	DAKO^2^

^1^Detects both wild-type and mutant p53 protein; ^2^DAKO, Glostrup, Denmark; ^3^The SP17 antibody kindly provided by Dr. Maurizio Chiriva, Texas Tech University; ^4^Cell Signaling, Danvers, USA; ^5^Zymed, San Francisco, USA; ^6^The HCA2 and HC-10 antibodies were a gift from Professor Dr. Neefjes, Netherlands Cancer Institute, Amsterdam, The Netherlands.

**Table 2:** Patient and tumor characteristics.

	All patients (*n* = 270)
*Age (years) *	
Mean (SD)	56.9 (13.8)
	* n* (%)
FIGO* stage	
Stage I	67 (24.9)
Stage II	26 (9.7)
Stage III	144 (53.5)
Stage IV	32 (11.9)
Missing	1

*Tumor type*	
Serous	147 (59.8)
Mucinous	37 (15.0)
Endometrioid	42 (17.1)
Clear cell	17 (6.9)
Undifferentiated	3 (1.2)
Missing	24

*Differentiation grade*	
Grade I	51 (20.2)
Grade II	77 (30.6)
Grade III	113 (44.8)
Undifferentiated	11 (4.4)
Missing	18

*Residual disease*	
<2 cm	155 (59.0)
≥2 cm	94 (35.7)
Positive**	21 (5.3)

*FIGO: International Federation of Gynecology and Obstetrics. **Amount unknown.

**Table 3:** Expression levels of antigen and MHC class I components.

	P53^1^	SP17^1^	Survivin^1^	WT1^1^	NY-ESO-1^1^
	*n *(%)	*n *(%)	*n *(%)	*n *(%)	*n *(%)
Normal/negative	130 (52.0)	78 (31.1)	23 (10.0)	100 (43.7)	219 (89.0)
Overexpression/positive	120 (48.0)	173 (68.9)	208 (90.0)	129 (56.3)	27 (11.0)
Missing	20	19	39	41	24

	HLA*- *A^+^ */ * *β*_2_ *- *m^+2^	HLA*-*B*/ *C^+^ */ * *β*_2_ *- *m^+2^	MHC class I^3^		
Positive^4^	98 (53.6)	136 (74.7)	143 (78.1)		
Negative^4^	85 (46.4)	46 (25.3)	40 (21.9)		
Missing		1			

^1^primary EOC patients,* n* = 270; ^2^staining in subgroup, *n* = 183; ^3^MHC class I expression is defined as HLA-A and *β*_2_-m and/or HLA-B/C and *β*_2_-m coexpression; ^4^positive is both components HLA-A/B/C and *β*_2_-m expressed, negative are all other phenotypes.

**Table 4:** Expression of single or multiple antigens in EOC.

Number of antigens	*n*	%	Cumulative %
1	40	15.2	15.2
2	70	26.6	41.8
3	70	26.6	68.4
4	58	22.1	90.5
5	7	2.7	93.2
None	18	6.8	100.0
Missing	7		

*n* = 270.

**Table 5:** Expression of specific antigen combinations in EOC.

Antigen combinations	% (*n*/total)
*One antigen*	
p53	48.0 (120/250)
SP17	68.9 (173/251)
Survivin	90.0 (208/231)
WT1	56.3 (129/229)
NY-ESO-1	11.0 (27/246)

*Two antigens*	
p53, SP17	84.2 (203/241)
p53, survivin	95.5 (214/224)
p53, WT1	73.1 (163/223)
p53, NY-ESO-1	52.7 (125/237)
SP17, surviving	94.7 (215/227)
SP17, WT1	82.3 (186/226)
SP17, NY-ESO-1	74.0 (179/242)
survivin, WT1	93.0 (212/228)
survivin, NY-ESO-1	90.8 (208/229)
WT1, NY-ESO-1	60.3 (138/229)

*Three antigens*	
p53, SP17, survivin	97.7 (217/222)
p53, SP17, WT1	91.4 (202/221)
p53, SP17, NY-ESO-1	86.4 (203/235)
p53, survivin, WT1	95.9 (213/222)
p53, survivin, NY-ESO-1	95.5 (213/223)
p53, WT1, NY-ESO-1	74.4 (166/223)
SP17, survivin, WT1	96.0 (216/225)
SP17, survivin, NY-ESO-1	95.6 (216/226)
SP17, WT1, NY-ESO-1	84.5 (191/226)
survivin, WT1, NY-ESO-1	93.4 (213/228)

*Four antigens*	
p53, SP17, survivin, WT1	98.2 (216/220)
p53, SP17, survivin, NY-ESO-1	97.7 (216/221)
p53, SP17, WT1, NY-ESO-1	92.3 (204/221)
p53, survivin, WT1, NY-ESO-1	95.9 (213/222)
SP17, survivin, WT1, NY-ESO-1	96.4 (217/225)

*All antigens*	
p53, SP17, survivin, WT1, NY-ESO-1	98.2 (216/220)

*n* = 270.

**Table 6:** Coexpression of MHC class I components with tumorantigens.

	p53^+^	SP17^+^	Survivin^+^	WT1^+^	NY-ESO1^+^	Tumorantigen^+2^
	*n *= 80	*n *= 116	*n *= 152	*n *= 89	*n *= 20	*n* = 173
	*n*/total (%)	*n*/total (%)	*n*/total (%)	*n*/total (%)	*n*/total (%)	*n*/total (%)
*HLA-* *A* ^+^ *β*_2_ *m* ^+^	52/80	64/116	80/152	50/89	13/20	96/173
*n *= 98	(65.0)	(55.2)	(52.6)	(56.2)	(65.0)	(55.0)
*HLA-B/* *C* ^+^ *β*_2_ *m* ^+^	62/78	94/116	111/151	67/89	15/20	129/171
*n *= 136	(79.5)	(81.0)	(73.5)	(75.3)	(75.0)	(75.4)
*MHC class * *I* ^+^	66/80	96/116	117/152	72/89	16/20	136/173
*n* = 143	(82.5)	(82.8)	(77.0)	(80.9)	(80.0)	(78.4)
*All* *p* *a* *t* *i* *e* *n* *t* *s* ^1^	66/174	96/178	117/169	72/167	16/175	136/183
*n* = 183	(37.9)	(53.9)	(69.2)	(43.1)	(9.1)	(74.3)

^1^Antigen^+^ and MHC class I^+^ in all subgroup patients (*n *= 183); ^2^ ≥ 1 tumorantigen expression.
